# An economic feasibility assessment of implementing photovoltaic module reflectors under Malaysian meteorological conditions

**DOI:** 10.1038/s41598-024-54031-x

**Published:** 2024-02-09

**Authors:** Sakhr M. Sultan, M. Z. Abdullah, C. P. Tso, N. F. Nik Abllah, N. Zakaria, Raheem K. Ajeel, K. Sobayel

**Affiliations:** 1 Solar Energy Research Institute, Universiti Kebangsaan Malaysia, 43600 Bangi, Selangor Malaysia; 2 School of Mechanical Engineering, Universiti Sains Malaysia, 14300 Nibong Tebal, Pulau Pinang Malaysia; 3 Faculty of Engineering and Technology, Multimedia University, Jalan Ayer Keroh Lama, 75450 Melaka, Malaysia; 4 Perunding Pinang Sdn. Bhd., Suite B, Top Floor University Height, 83D-3-17, Jalan Sungai Dua, 11700 Pulau Pinang, Malaysia; 5 Department of Sewerage Services, Ministry of Environment and Water, Blok B, Aras 2 Dan 3, Suasana PjH, No. 2, Jalan Tun Abdul Razak, Presint 2, 62100 Putrajaya, Malaysia

**Keywords:** Solar energy, Photovoltaic module, Reflector, Output energy, Cost effectiveness, Photovoltaic efficiency, Energy science and technology, Engineering

## Abstract

The use of a reflector can increase the solar radiation on the photovoltaic module (PV) surface, whereby the energy output can be improved. However, the economic feasibility may need to be considered too. This study is conducted, for the first time, due to the lack of studies regarding the economic feasibility assessment of implementing reflectors under the Malaysian meteorological conditions. The outcome will give information about the suitability for implementing a PV reflector in Malaysia through an experimental setup at a sewage treatment site, for two months in 2022. The Malaysian meteorological data, which include daily solar radiation, ambient temperature and wind velocity, were collected to study the output energy, efficiency and the economic perspective of a PV. In February 2022, the PV was operating without a reflector and the averaged values for the monthly solar radiation, ambient temperature and wind velocity were 539.9 MJ/m^2^, 28.4 °C and 2.2 m/s, respectively, which resulted in an output energy of 106.43 kWh. On the other hand, for April 2022, the PV was operating with a reflector. With the respective averaged input parameters 544.98 MJ/m^2^, 28.9 °C and 1.51 m/s, the output energy was 121.94 kWh. It is thus shown that the PV with a reflector increases the PV’s output energy by 14.57%. Also, it is shown that the cost-effective factor value is 0.955 which means that the PV reflector is economically feasible to be implemented under the Malaysian meteorological conditions. Hence, extensive research should be conducted to improve the performance of PV reflectors. The findings of this paper maybe useful for researchers and/or manufacturers of PV reflectors.

## Introduction

National per capita energy consumption is often regarded as a measure of economic growth. These days, energy is not only seen as a production input but also as a strategic concept that shapes global politics and economics and establishes the framework for international interactions. The need for energy is growing, but there are only few energy sources available. Furthermore, there is an uneven distribution of energy resources among the nations which holds true for both the levels of consumption and the energy reserves. This makes it extremely difficult for nations with limited energy resources to obtain these resources under fair, dependable, and sustainable circumstances, in order to fulfill their rising energy needs. Research is being done to discover ways to combat the energy scarcity. Since the sun does not belong to any one entity and produces energy that is roughly 10,000 times greater than that of fossil fuels, hydroelectric power, and nuclear power, solar energy may be the energy of the future. There is still space for development even with its poor conversion rate^1^.

**Figure 1:**
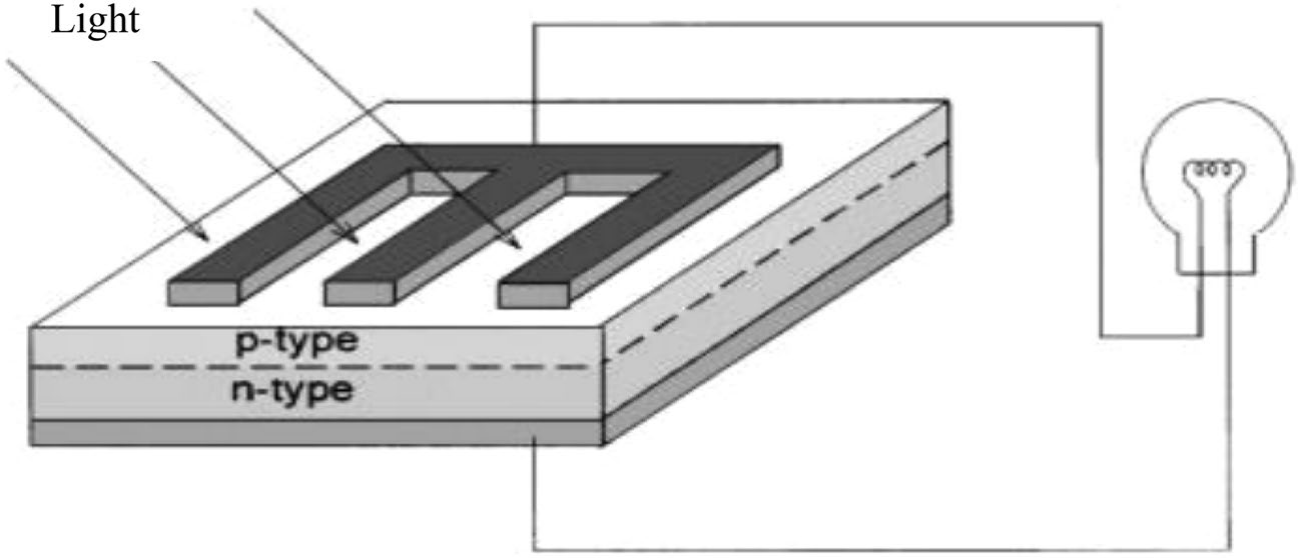
Sunlight is transformed to electric current flowing through a load^3^.

Solar energy encompasses two main technologies: photovoltaic technology and solar thermal collectors. Photovoltaic technology converts solar radiation into electricity through the photovoltaic effect, a process characterized by the generation of a self-produced electromagnetic field and current, capable of supplying power to a load, as depicted in Fig. 1^2,3^. Then, solar thermal collectors are used for heating applications^4–6^.

One of the available solutions to enhance the PV performance is to integrate a reflector^7^. The major benefits of deploying a reflector are as the following^8–11^:

- Energy efficient PV.
- Reducing the number of PVs for the same output energy.
- Reducing cost.
- Reducing the carbon footprint.

**Figure 2:**
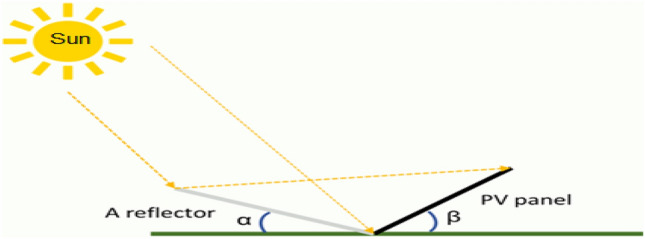
A schematic diagram for a PV with a reflector^19^.

The general design of a solar cell aims to maximize the absorption of incoming sunlight to generate electricity efficiently. The active layer of a solar cell, typically made of semiconductor material like silicon, is where the absorption of photons and generation of electron–hole pairs occur. This layer is engineered to have properties that optimize light absorption within a certain range of wavelengths corresponding to the solar spectrum. However, even with a well-designed absorption layer, not all incident light is effectively absorbed. Some of it may be reflected off the surface of the cell due to differences in refractive indices between the semiconductor material and the surrounding medium (usually air). This reflected light represents a loss of potential energy conversion. To minimize this loss, a surface reflector can be added to the design of the solar cell. This reflector is typically a thin layer with a high refractive index material deposited on the surface of the semiconductor material. According to Snell's law, when light passes from one medium to another with a different refractive index, it undergoes refraction and reflection. By strategically choosing the thickness and material of the surface reflector, it's possible to reduce the amount of light that is reflected back into the air and increase the amount that is transmitted into the active layer for absorption. In summary, while the absorption layer of a solar cell is designed to maximize light absorption, the addition of a surface reflector based on Snell’s law helps to minimize losses due to surface reflection thereby enhancing the overall efficiency of the solar cell. Also, the use of reflector is beneficial when the amount of solar radiation is low. Because it helps to maximize the amount of solar radiation that strikes the solar cell, improving the electrical current production^12^. Figure 2 illustrates a schematic diagram of a PV with a reflector. Depending on the weather conditions and the type of the reflector used, the PV performance can be improved^13–15^. Several studies were conducted for the purpose of improving the PV performance as summarized in Table 1. The output power of a PV integrated V-trough concentrator was obtained numerically and experimentally under outdoor operating conditions^16^. Results showed that the maximum power improvement was 31.2%. In a separate investigation, an experiment was undertaken to explore the impact of reflector parameters on output power^13^. The findings demonstrated that reflectors could augment output power by as much as 60%. Another study involved the construction of a PV with an aluminum sheet reflector, resulting in a 15% increase in output power^14^. An experimental inquiry into a PV system with a concentrator revealed a significant 48% rise in output power^17^. Furthermore, an experimental and economic analysis of a PV system incorporating a cooler and a reflector indicated an enhanced PV efficiency of 10.68%, with a payback time of 4.2 years^18^. A numerical examination of a PV utilizing an aluminum sheet explored the impact of tilt angle on PV efficiency, demonstrating performance improvement with an increase in tilt angle. The maximum PV efficiency, obtained at an optimum tilt angle of 75 degrees, was 19%^19^. Introducing a novel concept, a PV featuring a curved reflector was proposed^20^, resulting in a notable increase in the spatial solar power of the system to 61%. Subsequent to a numerical and experimental analysis of a PV system incorporating both a reflector and a cooler, the PV efficiency was observed to rise to 36%^21^. Additionally, a three-dimensional model was introduced for a PV system with a stainless steel (SS) component, aiming to elevate PV efficiency to 34.16%^22^.

**Table 1:** Previous studies on the existing PV with reflectors.

Reference	Brief system description	Power improvement percentage, %	Mode of study
^13^	The experimental investigation focused on examining how the parameters of the reflector affected the efficiency of PV	60	Numerical and experimental study
^14^	A numerical and experimental analysis was conducted on a PV equipped with a reflector made of aluminum sheet	15	Numerical and experimental study
^16^	A predictive model was introduced for forecasting the output power of a V-trough concentrator equipped PV	31.2	Numerical study
^17^	An experimental investigation was conducted on a PV incorporating a V-trough concentrator under outdoor operating conditions	48	Experimental study
^18^	An experimental study was conducted on PV featuring both a reflector and a cooler	10.68	Experimental study
^19^	A PV with a reflector underwent analytical examination	19	Numerical study
^20^	A new curved reflector for PV was proposed	61	Numerical and experimental study
^21^	A PV with flat reflector and cooler was theoretically and experimentally studied	36	Numerical and experimental study
^22^	A technique for enhancing PV performance through the incorporation of a commercially available flat stainless-steel reflector is identified	34.16	Numerical and experimental study
^23^	Mirror reflectors on PV modules during both summer and winter seasons were investigated	Up to 20%	Numerical and experimental study
^24^	A phase change material (PCM) and mirror reflectors in conjunction with PV modules at various angles were explored	12.5	Numerical and experimental study
^28^	An experimental investigation on the impact of reflectors attached to a PV panel cooled through a natural cooling approach was conducted	Up to 11.87%	Experimental study
^29^	The effects of PV/TEG and reflectors on cooling a PV system were studied	4	Numerical and experimental study
^30^	A linear Fresnel reflector coupled to PV panels was investigated	14.7	Numerical and experimental study

Malik and Chandel^23^ investigated the influence of mirror reflectors on PV modules during both summer and winter seasons. Their findings indicated a notable enhancement in the power output of the PV, showing an improvement ranging from 10 to 20% in both seasons.

Lotfi et al.^24^ explored the performance of phase change material (PCM) and mirror reflectors in conjunction with PV modules at various angles, revealing a notable increase in the power output of PCM/reflector by approximately 12.5%. Elqady et al.^25^, using a numerical approach, evaluated the effects of integrating reflectors and double-layer microchannel heat sinks on a PV panel, suggesting a potential temperature reduction of 3–4% under varying concentration ratios. Paul et al.^26^ introduced booster reflectors as a means to augment the performance of PV modules. Michael et al.^27^ developed V-shape concentrators positioned at a 130° angle to PV modules, resulting in a temperature reduction of 4.95 °C for the cooled PV module, as indicated by their results. Kabeel et al.^28^ conducted experimental investigations on the impact of reflectors attached to a PV panel cooled through a natural cooling approach. Their findings highlighted an improvement in the electrical efficiency of the PV panel by about 9.71–11.87%. Shoguchkarov et al.^29^, employing both numerical and experimental methods, assessed the effects of PV/TEG and reflectors on cooling a PV system, demonstrating an approximate 4.8% improvement in output power. Wang et al.^30^ constructed and experimentally examined linear Fresnel reflectors coupled to PV panels, reporting a notable 14.7% enhancement in the efficiency of the PV panel. The impact of nanofluid spectral splitter on efficiency of concentrated PV thermal system was studied^31^. Results showed that the cell temperature decreases about 47.82% with applying the filter and the electrical performance was 25.09% with new cooling unit. The impact of dust accumulation on concentrated PV equipped with thermoelectric layer in presence of nanofluid flow within porous heat sink was studied^32^. It was found that the thermoelectric and PV efficiencies decrease by 12.11% and 26.47% with the rise of dust.

On the other hand, the economic feasibility assessment for PV enhancers can be performed using the PV enhancer cost effectiveness factor, F_CE_, which is an important parameter that is needed to be taken into account when designing an enhancer (reflector/cooler) for the PV^33^. This parameter states if the designed reflector/cooler has added a sufficient power to the PV as compared to its cost or getting additional PV is better. F_CE_ was denoted as^33^

1 $${{\text{F}}}_{{\text{CE}}}= \frac{{{\text{P}}}_{{\text{PV}},{\text{out}}}+\frac{\mathrm{ Z}}{{\text{Y}}}}{{{\text{P}}}_{{\text{PVCE}},{\text{out}}}},$$

where ${{\text{P}}}_{{\text{PV}},{\text{out}}}$ is the gained power from a PV without an enhancer and ${{\text{P}}}_{{\text{PVCE}},{\text{out}}}$ is the gained power from a PV with an enhancer. Y and Z are the cost of one watt of PV power and the cost of the enhancer, respectively.

F_CE,min_ is the minimum value of F_CE._ It shows that the PV enhancer has reached the optimum performance and can be denoted as^33^

2 $${{\text{F}}}_{{\text{CE}},{\text{min}}}= \frac{{{\text{P}}}_{{\text{PV}},{\text{out}}}}{{{\text{P}}}_{{\text{PVCE}},{\text{max}}}},$$

where ${{\text{P}}}_{{\text{PVCE}},{\text{max}}}$ is the maximum output power from a PV with an enhancer.

Three classifications of F_CE_ are given, as the following^33^:

1. If ${{\text{F}}}_{{\text{CE}},{\text{min}}}{\le {\text{F}}}_{{\text{CE}}}<1,$ it denotes that the PV enhancer is economically feasible.
2. If ${{\text{F}}}_{{\text{CE}}}=1$, it denotes that the PV enhancer is neutral.
3. If $1{<{\text{F}}}_{{\text{CE}}}<\infty$, it denotes that the PV enhancer is not economically feasible.

Equation (1) can be modified as the following^34^

3 $${{\text{F}}}_{{\text{MCE}}}= \frac{{{\text{n}}\times {\text{P}}}_{{\text{cell}},{\text{out}}}+\frac{\mathrm{ Z}}{{\text{Y}}}}{{{\text{P}}}_{{\text{PVCE}},{\text{out}}}},$$

where is the modified PV enhancer cost effectiveness factor, n is the number of solar cells for a PV with an enhancer and ${{\text{P}}}_{{\text{cell}},{\text{out}}}$ is the power from one solar cell without an enhancer.

Hence, this study is proposed to serve researchers and/or manufacturers by filling the research gap found in reporting the economic feasibility assessment of implementing reflector under Malaysian meteorological conditions. The experimental work is conducted at a sewage treatment site in Malaysia. The testing period was for 2 months in 2022 which are February and April. The PV was operating without and with a reflector, in February and April, respectively. The output energy and efficiency are reported for the examined systems. Economic analysis is conducted to determine the suitability to use the reflector technology in Malaysia. It will be shown that the reflector increases the PV’s output energy and is economically feasible to be implemented under the Malaysian meteorological conditions.

## Research methodology and experimental procedure

**Figure 3:**
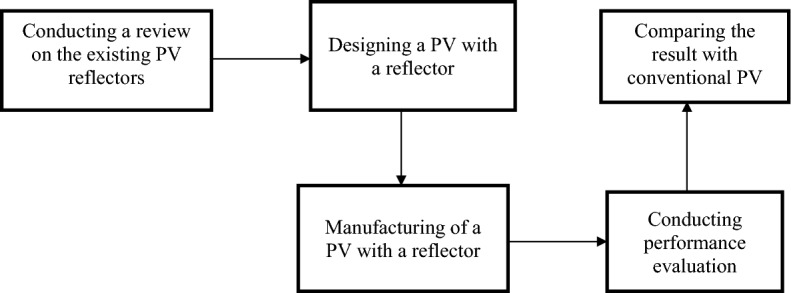
The research activities performed in this study.

**Figure 4:**
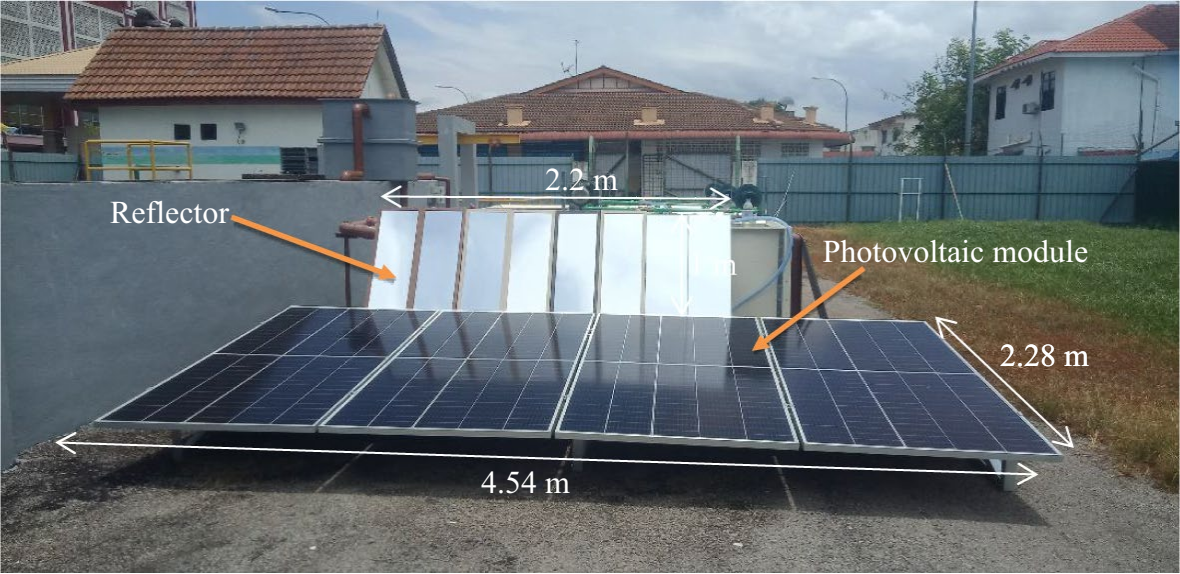
The experimental set up for the PV with a reflector.

Figure 3 shows a block diagram of the research methodology conducted in this study. Firstly, extensive research on the existing PV with reflectors is performed to find the research gap. Then, a complete knowledge is gained which leads to studying the economic feasibility of implementing reflectors under the Malaysian climatic conditions. The second step is to design the PV with a reflector. In the present study, a mirror is used as a reflector of length of 2.2 m and width of 1 m. The third step is initiated to manufacture the designed system as shown in Fig. 4. The fourth step is introduced for performing system testing over a range of operating conditions. The fifth step is indicated for comparing the PV with a reflector with the conventional PV to investigate the system performance enhancement and the economic feasibility assessment. Also, there is a checking on the PV temperature from time to time to confirm that the PV is operating within the temperature range set by the manufacturer. The specifications of the PV and list of items used in the current study are shown in Tables 2 and 3, respectively. In February, the PV was operating without a reflector, and in April, a reflector was attached to the PV. The Malaysian Meteorological Department (MMD) provided essential meteorological information, such as daily solar radiation, ambient temperature, and wind velocity, aiding in the examination of the output energy, efficiency, and the economic feasibility assessment of PV reflectors.

**Table 2:** The specifications of the PV used in the current study.

Parameter	Value
Maximum power (P_max_)	525 Wp
Voltage at maximum power (V_mpp_)	41.15 V
Current at maximum power (I_mpp_)	12.76 A
Open circuit voltage (V_oc_)	49.15 V
Short circuit current (I_sc_)	13.65 A
Panel efficiency	20.3%
Temperature coefficient of P_max_	− 0.35%/°C
Operating temperature range	− 40 to 85 °C
Panel dimensions (length × width × thickness)	2279 mm × 1134 mm × 3.5 mm

**Table 3:** List of components used in the present study.

No.	Item	Quantity
1.	PV	1
2.	Reflector	1
3.	Solar power meter	1
4.	Data logger	1
5.	Connector	1
6.	Multimeter	1

### Uncertainty analysis

The precision of instrument readings influences the experimental error associated with independent variables, such as electrical current (I) and voltage (V). The experimental error for the dependent variable (power) can be determined from the experimental errors of the independent variables using the following formula.

4 $${\text{U}}=\sqrt{{\left(\frac{\partial {\text{P}}}{\partial {\text{V}}}.{{\text{U}}}_{{\text{V}}}\right)}^{2}+{\left(\frac{\partial {\text{P}}}{\partial {\text{I}}}.{{\text{U}}}_{{\text{I}}}\right)}^{2}},$$

where

5 $${\text{P}}={\text{V}}\times {\text{I}}.$$

As per the manufacturer of the multimeter, both and exhibit an average accuracy of 0.30% each. Consequently, the experimental error for power is calculated to be 2.80%.

## Results and discussions

The experimental observations are explored for a PV under both natural conditions and with the incorporation of a reflector. The study considers the impact of local climatic factors, including ambient temperature, solar radiation, and wind velocity, on the PV’s output energy, efficiency, and economic viability. A comparative analysis between the PV with and without a reflector is undertaken. The findings indicate that the PV system with a reflector outperforms its counterpart without a reflector and is economically viable for application in Malaysian climatic conditions.

### The daily solar radiation, ambient temperature and wind velocity

**Figure 5:**
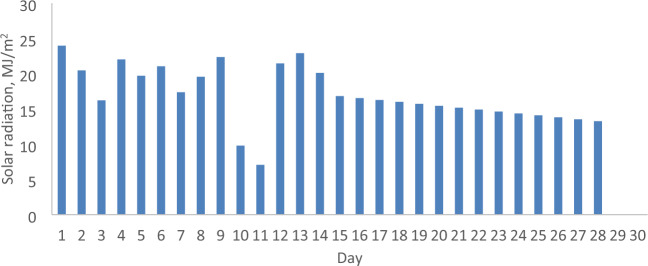
The daily solar radiation for February 2022.

**Figure 6:**
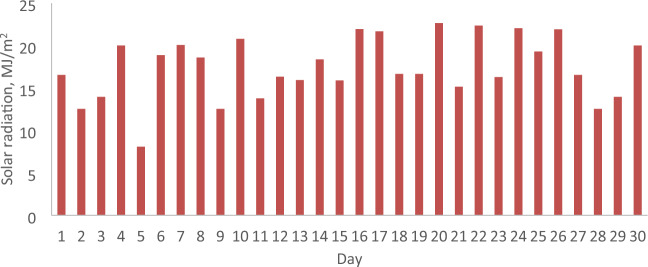
The daily solar radiation for April 2022.

**Figure 7:**
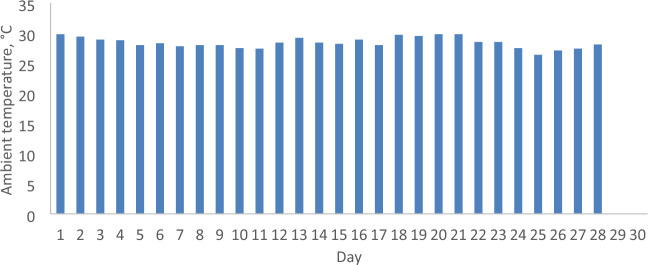
The daily ambient temperature for February 2022.

**Figure 8:**
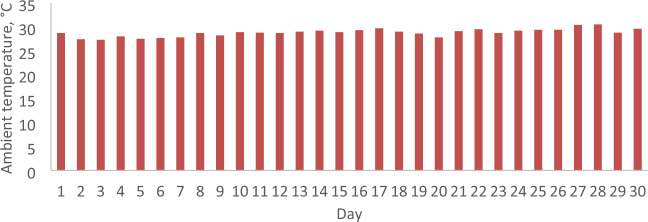
The daily ambient temperature for April 2022.

**Figure 9:**
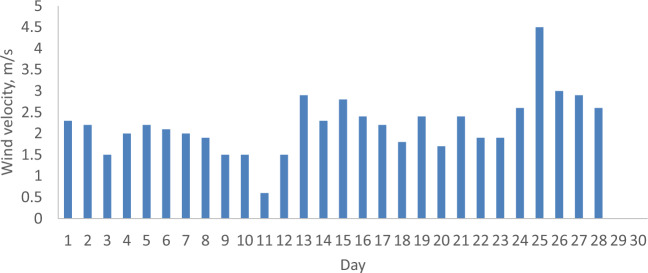
The daily wind velocity for February 2022.

**Figure 10:**
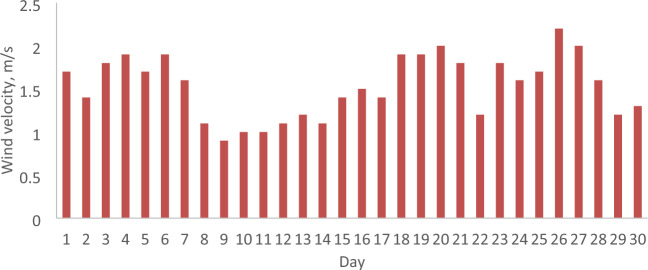
The daily wind velocity for April 2022.

The parameters relevant to the months of February and April 2022 in Malaysia, including daily solar radiation, ambient temperature, and wind velocity, are analyzed to assess the economic implications of incorporating a reflector in a PV. These parameters serve as the operational conditions for both the PV with and without a reflector. Figures 5, 6, 7, 8, 9 and 10 depict the operational conditions (solar radiation, ambient temperature, and wind velocity) for February and April 2022. For February 2022, the minimum values for daily solar radiation, ambient temperature, and wind velocity are 7.07 MJ/m^2^, 26.4 °C, and 0.6 m/s, respectively, while the maximum values are 23.96 MJ/m^2^, 29.8 °C, and 2.3 m/s, respectively. The differences between the maximum and minimum values for solar radiation, ambient temperature, and wind velocity are 16.89 MJ/m^2^, 3.4 °C, and 1.7 m/s, respectively, which are expected to influence the performance of both the PV with and without a reflector. The average daily values for solar radiation, ambient temperature, and wind velocity are 16.94 MJ/m^2^, 28.4 °C, and 2.2 m/s, respectively. Figures 5 and 6 also indicate total solar radiation values for February and April 2022 being 539.98 and 544.98 MJ/m^2^, respectively. Notably, the solar radiation in April 2022 surpasses that in February 2022.

Conversely, in April 2022, the analysis reveals that the minimum values for daily solar radiation, ambient temperature, and wind velocity are 8.08 MJ/m^2^, 27.3 °C, and 0.9 m/s, respectively, as illustrated in Figs. 5, 6, 7, 8, 9 and 10. In contrast, the maximum values for these parameters are 22.65 MJ/m^2^, 30.5 °C, and 2.2 m/s, respectively. The disparities between the maximum and minimum values for solar radiation, ambient temperature, and wind velocity are 14.57 MJ/m^2^, 3.2 °C, and 1.3 m/s, respectively. Additionally, the average daily values for solar radiation, ambient temperature, and wind velocity are observed to be 21.36 MJ/m^2^, 28.8 °C, and 1.97 m/s, respectively.

### The daily output energy from the PV with and without a reflector

**Figure 11:**
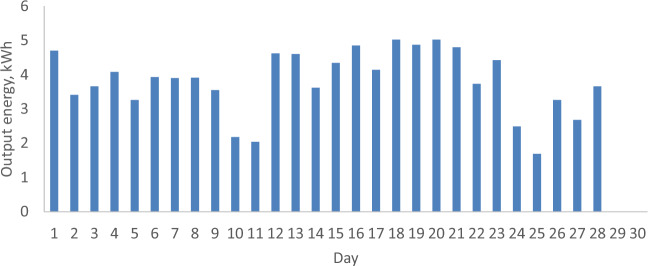
The daily output energy from PV without a reflector for February 2022.

**Figure 12:**
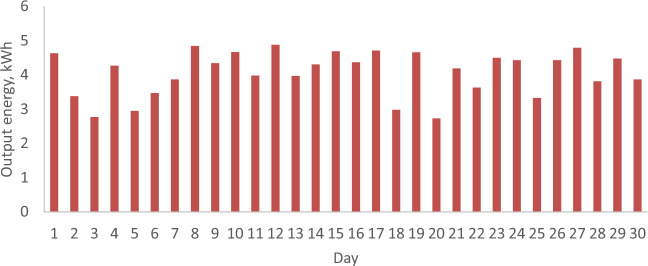
The daily output energy from PV with a reflector for April 2022.

Figures 11 and 12 illustrate the daily energy output from the PV both with and without a reflector during February and April 2022 in Malaysia. Generally, a direct correlation between output energy and solar radiation is observed, as the output energy increases in tandem with solar radiation due to its proportional relationship with the short circuit current. Examining Figs. 11 and 12 for February 2022, when the PV lacks a reflector, a notable reduction in output energy occurs as solar radiation drops from 23.96 (Day 1) to 13.53 MJ/m^2^ (Day 27), resulting in a decrease from 4.63 to 2.68 kWh, respectively. In contrast, for April 2022, when PVs are equipped with a reflector, the output energy rises from 2.95 to 4.43 kWh with an increase in solar radiation from 8.08 (Day 5) to 21.92 MJ/m^2^ (Day 24). It’s worth noting that the total output energy for February and April 2022 is 106.43 and 121.94 kWh, respectively. Despite the higher wind velocity in February, which can lower PV temperature and enhance performance, the PV without a reflector installed in February exhibits inferior performance compared to the PV with a reflector installed in April. This is evident in the higher output energy in April, resulting in a 14.57% increase. These findings highlight that, under Malaysian climatic conditions, PVs with reflectors demonstrate superior performance compared to those without.

### The daily PV efficiency from PV with and without reflector

**Figure 13:**
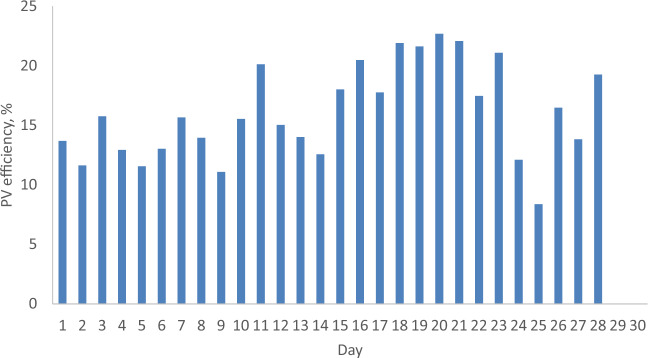
The daily efficiency from a PV without and with reflector for February 2022.

**Figure 14:**
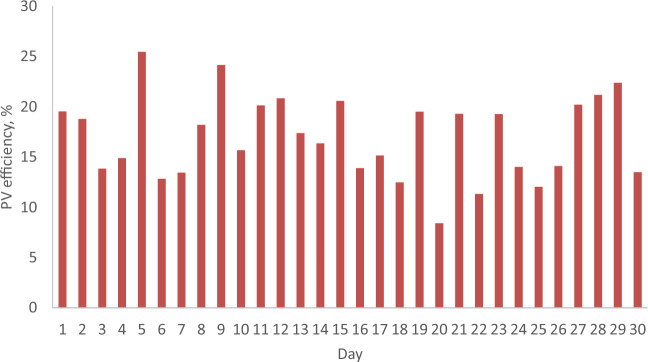
The daily efficiency from a PV without and with reflector for April 2022.

Figures 13 and 14 depict the daily efficiency of a PV with and without a reflector during February and April 2022. The efficiency of the PV increases in tandem with solar radiation due to its proportional relationship with output power. According to Figs. 13 and 14, the maximum PV efficiency is 25.5% for the PV with a reflector, surpassing the 22.7% efficiency for the PV without a reflector by 2.8%. Notably, the PV efficiency for the reflector-equipped PV exceeds the efficiency at Standard Test Conditions (STC), which is 20.3%, attributable to the higher solar radiation values. Conversely, the minimum PV efficiency is 14.89%, compared to 12.93% for the PV without a reflector, resulting in a 1.96% difference. This indicates that the PV with a reflector demonstrates superior performance compared to the PV without a reflector under Malaysian meteorological conditions.

### Special cases

Table 4 shows the special cases where the reported PV efficiency was higher in February as compared to April 2022. It is because the ambient temperature in February is lower compared to the ambient temperature in April. Also, the wind velocity in February is higher as compared to the wind velocity in April. From Table 4, it is seen that in Day 16, 17 and 23 of February 2022, the PV efficiency values are 20.5, 17.76 and 16.5%. On the other hand, they are 13.89, 15.15 and 14.1% for the same days of April 2022. From above results, it can be seen that the PV efficiency in February, when the PV was operating without a reflector, is higher as compared to the PV efficiency for a PV with a reflector that was operating in April 2022, because the ambient temperature in February was lower than the ambient temperature in April, as well as the wind velocity in February was higher as compared to the wind velocity in April. The ambient temperature and wind velocity values in February for Day 16, Day 17 and Day 26 are 28.9 °C and 2.4 m/s, 28 °C and 2.2 m/s, 27.1 °C and 3 m/s, respectively. In April, their values are 29.3 °C and 1.5 m/s, 29.7 °C and 1.4 m/s, 29.4 °C and 2.2 m/s, respectively. On the other hand, in Day 7, 19, 21, 22 and 23, the PV efficiency in February was higher as compared to the PV efficiency in April, because the wind velocity in February was higher as compared to the wind velocity in April. In February, the PV efficiencies are 15.66, 21.63, 22.67, 17.46 and 21.1%, while they are 13.45, 19.5, 19.3, 11.32 and 19.27% in April for Day 7, 19, 21, 22 and 23, respectively. It is concluded that the operating conditions are having a direct influence on the PV efficiency.

**Table 4:** The special cases where the reported PV efficiency was higher in February as compared to April 2022.

Day	February 2022			April 2022			Remark
	PV efficiency (%)	Ambient temperature (°C)	Wind velocity (m/s)	PV efficiency (%)	Ambient temperature (°C)	Wind velocity (m/s)	
3	15.74	28.9	1.5	13.84	27.3	1.8	–
7	15.66	27.8	2.0	13.45	27.8	1.6	Wind velocity in February is higher than April wind velocity
16	20.5	28.9	2.4	13.89	29.3	1.5	Wind velocity in February is higher than April wind velocity. Ambient temperature in February is lesser than April ambient temperature
17	17.76	28.0	2.2	15.15	29.7	1.4	Wind velocity in February is higher than April wind velocity. Ambient temperature in February is lesser than April ambient temperature
18	21.9	29.7	1.8	12.47	29.0	1.9	–
19	21.63	29.5	2.4	19.5	28.6	1.9	Wind velocity in February is higher than April wind velocity
20	22.68	29.8	1.7	8.41	27.8	2	–
21	22.07	29.8	2.4	19.3	29.1	1.8	Wind velocity in February is higher than April wind velocity
22	17.46	29.8	1.9	11.32	29.5	1.2	Wind velocity in February is higher than April wind velocity
23	21.1	28.7	1.9	19.27	28.7	1.8	Wind velocity in February is higher than April wind velocity
26	16.5	27.1	3	14.1	29.4	2.2	Wind velocity in February is higher than April wind velocity. Ambient temperature in February is lesser than April ambient temperature

### The economic feasibility assessment for a PV with a reflector

Now, to perform the economic feasibility assessment for a PV with a reflector, the cost effectiveness factor (F_CE_) should be calculated, based on Eq. (1). Given that the cost of one watt of PV power is RM25. Table 5 includes the parameters needed to perform the cost economic feasibility analysis for a PV with a reflector.

**Table 5:** The economic feasibility assessment for a PV with a reflector.

Item	P_PV, out_, kW	P_PVWE, out_, kW	One watt of PV power cost, RM*	PV enhancer Cost, RM*	F_CE_	Economic feasibility remark
PV with a reflector	106.43	121.94	25	250	0.955	Economically feasible

*Malaysian Ringgit.

From Table 5, the PV with a reflector has F_CE_ value of 0.955. It shows that if the cost of the reflector is converted to PV power, and then this power is added to the P_PV,out_, it will be lesser than the output power from the PV with a reflector. From the above results, it shows that the PV with a reflector is economically feasible to be applied under Malaysian climatic conditions. Hence, extensive research should be conducted to improve the performance of PV reflectors.

### Comparison between existing and current work

The investigation of a passive cooling efficiency of a concentrated PV module, employing two distinct designs of innovative passive fin heat sinks: lapping and longitudinal was conducted^18^. A Design of Experiment (DOE) methodology was utilized to determine the optimal design parameters, encompassing fin height, fin pitch, fin thickness, number of fins, and tilt angle. The experimental trials were conducted under actual environmental conditions, employing the identified optimal design parameters for the passive fin heat sinks. Under an average solar irradiance of 1000 W/m^2^ and an ambient temperature of 33 °C, the findings indicated that passive cooling with lapping fins exhibited superior performance, resulting in a mean PV module temperature 24.6 °C lower than the reference PV module. Consequently, the achieved electrical efficiency and power output were notably higher at 10.68% and 37.1 W, respectively. Subsequently, a life cycle cost analysis (LCCA) was executed. The analysis revealed that the payback periods for PV modules with longitudinal, lapping fins, and bare PV modules are 4.2, 5, and 8.4 years, respectively. Consequently, the utilization of passive cooling techniques, particularly with the lapping fins design, was determined to be the preferred option for PV module cooling. The system was tested under Malaysian Meteorological conditions to validate the numerical results. The economic study of the existing work, using the cost effectiveness factor (F_CE_), cannot be assessed because the cost of one watt of PV power is not declared. In the present study, all the parameters needed to calculate F_CE_ are stated. Hence, the economic evaluation was performed.

## Conclusion

The utilization of a reflector is a technology aimed at augmenting solar radiation incident on the surface of a PV, leading to an enhancement in the PV energy output. However, there are instances where the adoption of this technology may not be economically viable, signifying that the cost of a given reflector outweighs the resultant increase in power generation. This study addresses the lack of research on the economic feasibility assessment of implementing reflectors under Malaysian meteorological conditions. To evaluate the suitability of deploying a PV reflector in Malaysia, an experimental study was conducted at a sewage treatment site over two months in 2022, specifically February and April. Meteorological data for Malaysia, encompassing daily solar radiation, ambient temperature, and wind velocity, was collected to analyze the PV’s energy output, efficiency, and economic viability. In February 2022, the PV operated without a reflector, recording monthly solar radiation, average monthly ambient temperature, and wind velocity values of 539.9 MJ/m^2^, 28.4 °C, and 2.2 m/s, respectively. Results indicated a monthly output energy of 106.43 kWh for the PV without a reflector. In contrast, during April 2022, the PV operated with a reflector, with input parameters of 544.98 MJ/m^2^, 28.9 °C, and 1.51 m/s for monthly solar radiation, ambient temperature, and wind velocity, respectively. The PV's monthly output energy with a reflector was 121.94 kWh. The findings demonstrated that the PV with a reflector increased output energy by 14.57% compared to the PV without a reflector. Additionally, the cost-effective factor was determined to be 0.955, indicating the economic feasibility of implementing a PV reflector under Malaysian meteorological conditions. Consequently, further research is recommended to enhance the performance of PV reflectors. The insights from this study can prove valuable for researchers and manufacturers involved in PV reflector technology.

## Data Availability

All data generated or analyzed during this study are included in this published article.

## Abbreviations

F: Factor, dimensionless
I: Current (A)
MMD: Malaysian Meteorological Department
n: Number of solar cell
P: Power (W)
RM: Ringgit Malaysia
STC: Standard test conditions
V: Voltage (V)
Y: One watt cost of PV power ($)
Z: Cost of manufacturing of PV enhancer ($)

## Subscripts

CE: Production cost effectiveness
cell: Solar cell
max: Maximum
MCE: Modified production cost effectiveness
min: Minimum
PV: Photovoltaic module
PVCE: Photovoltaic module enhancing technique
